# Differences in Cerebral Small Vessel Disease Magnetic Resonance Imaging Depending on Cardiovascular Risk Factors: A Retrospective Cross-Sectional Study

**DOI:** 10.3390/brainsci15080804

**Published:** 2025-07-28

**Authors:** Marta Ribera-Zabaco, Carlos Laredo, Emma Muñoz-Moreno, Andrea Cabero-Arnold, Irene Rosa-Batlle, Inés Bartolomé-Arenas, Sergio Amaro, Ángel Chamorro, Salvatore Rudilosso

**Affiliations:** 1 Faculty of Medicine and Health Sciences, University of Barcelona, Casanova 143, 08036 Barcelona, Spainsamaro@clinic.cat (S.A.);; 2 August Pi i Sunyer Biomedical Research Institute (IDIBAPS), Rosselló 149, 08036 Barcelona, Spain; laredo@recerca.clinic.cat (C.L.); cabero@recerca.clinic.cat (A.C.-A.); 3 Hospital Clínic Barcelona, Neurology Service, Villarroel 170, 08036 Barcelona, Spain; 4 Comprehensive Stroke Center, Department of Neuroscience, Hospital Clínic Barcelona, Villarroel 170, 08036 Barcelona, Spain

**Keywords:** cerebral small vessel disease, vascular risk factors, magnetic resonance imaging, spatial lesion analysis

## Abstract

**Background**: Vascular risk factors (VRFs) are known to influence cerebral small vessel disease (cSVD) burden and progression. However, their specific impact on the presence and distribution of each cSVD imaging marker (white matter hyperintensity [WMH], perivascular spaces [PVSs], lacunes, and cerebral microbleeds [CMBs]) and their spatial distribution remains unclear. **Methods**: We conducted a retrospective analysis of 93 patients with lacunar stroke with a standardized investigational magnetic resonance imaging protocol using a 3T scanner. WMH and PVSs were segmented semi-automatically, and lacunes and CMBs were manually segmented. We assessed the univariable associations of four common VRFs (hypertension, hyperlipidemia, diabetes, and smoking) with the load of each cSVD marker. Then, we assessed the independent associations of these VRFs in multivariable regression models adjusted for age and sex. Spatial lesion patterns were explored with regional volumetric comparisons using Pearson’s coefficient analysis, which was adjusted for multiple comparisons, and by visually examining heatmap lesion distributions. **Results**: Hypertension was the VRF that exhibited stronger associations with the cSVD markers in the univariable analysis. In the multivariable analysis, only lacunes (*p* = 0.009) and PVSs in the basal ganglia (*p* = 0.014) and white matter (*p* = 0.016) were still associated with hypertension. In the regional analysis, hypertension showed a higher WMH load in deep structures and white matter, particularly in the posterior periventricular regions. In patients with hyperlipidemia, WMH was preferentially found in hippocampal regions. **Conclusions**: Hypertension was confirmed to be the VRF with the most impact on cSVD load, especially for lacunes and PVSs, while the lesion topography was variable for each VRF. These findings shed light on the complexity of cSVD expression in relation to factors detrimental to vascular health.

## 1. Introduction

The term cerebral small vessel disease (cSVD) refers to a group of etiologically distinct pathologies that affect the small arteries, arterioles, capillaries, and venules in the brain [1]. The most prevalent type is type 1, also known as the “arteriosclerotic” or “hypertensive” type, which is strongly linked to age and vascular risk factors (VRFs) and is responsible for up to a quarter of ischemic strokes, mostly due to small perforating artery occlusions [2].

Neuroimaging studies are crucial for confirming the presence of cSVD, facilitating the evaluation of the extension and spatial distribution of the disease in the brain. Magnetic resonance imaging (MRI) is especially accurate at identifying the most typical cSVD lesions, which are white matter hyperintensities (WMHs), perivascular spaces (PVSs), lacunes, recent small subcortical infarcts (RSSIs), and cerebral microbleeds (CMBs), according to the STandards for ReportIng Vascular changes on nEuroimaging (STRIVE) initiative consensus [3].

**Figure 1:**
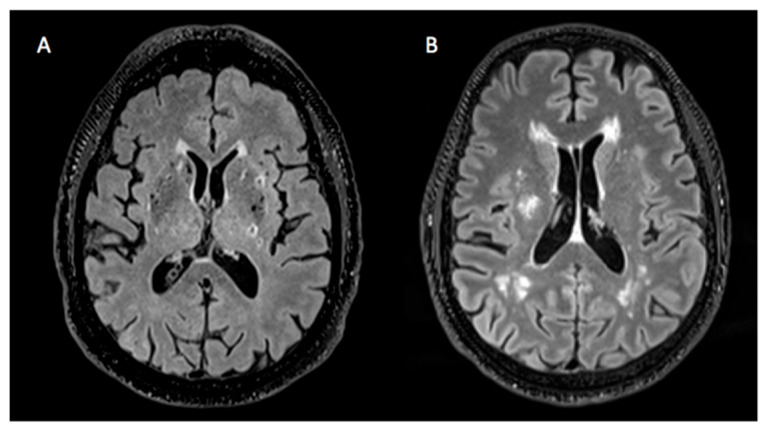
Heterogeneity of cSVD lesions. (**A**) Brain presenting a considerable amount of lacunes and hardly any WMH. (**B**) Brain showing a large quantity of WMH and no lacunes.

Although it is known that the global load of cSVD imaging markers is proportional to the presence of VRFs, the phenotype of cSVD lesions may differ according to specific VRFs. For example, some patients may present extended areas of WMH and few lacunes, or vice versa (Figure 1). In addition, certain imaging markers such as WMH, lacunes, and PVSs are known to exhibit distinct spatial distributions between patients with type 1 cSVD and those with cSVD of another etiology, such as cerebral amyloid angiopathy. However, the patterns of distribution for these individual imaging markers within type 1 cSVD in relation to the presence of VRFs are not well established [4].

Most of the cSVD burden can be explained by modifiable VRFs, which include hypertension, hyperlipidemia, diabetes, smoking, excessive alcohol consumption, obesity, poor diet, and cardiac causes [5]. Indeed, hypertension is known to be the main modifiable risk factor for cSVD. In Spain, hypertension affects approximately 33% of adults aged 30–79 years. Only 77% are aware of their condition, and 71% are receiving treatment, 64% of whom have their blood pressure (BP) under control [6]. Longitudinal imaging studies based on MRI demonstrated that BP-lowering treatments led to a reduction in WMH progression [7].

Hyperlipidemia’s prevalence in Spain among people aged 25–74 years is about 41%. Although hypolipidemic therapies are considered crucial for both primary and secondary prevention of cSVD [8], the evidence is low for their specific effect on WMH extension and progression [9].

Smoking has been associated with the increased presence of cSVD features in MRI in observational studies [5,10]. The impact of smoking on each cSVD marker is largely unknown.

Patients with diabetes have a higher risk of presenting cSVD [11], although the efficacy of glucose-lowering interventions for detaining cSVD progression is still unclear.

According to the latest clinical guidelines [12], identifying VRFs is crucial for preventing the progression of cSVD and serious clinical manifestations such as stroke and dementia, particularly through lifestyle interventions along with targeted treatments. In this context, understanding the association between VRFs and cSVD spatial patterns may enhance our knowledge of the underlying pathophysiological processes. Furthermore, identifying various patterns of cSVD lesions may help determine which factors should be prioritized for effective management.

The aim of this study was to assess the independent associations between four prevalent VRFs (hypertension, hyperlipidemia, diabetes, and smoking) with differential cSVD lesion load and spatial distribution patterns of WMH, PVSs, lacunes, and CMBs in patients with lacunar strokes. More specifically, we assessed the correlation between each VRF and the load of each cSVD lesion, as well as the distribution of these lesions according to the presence of each VRF.

## 2. Materials and Methods

### 2.1. Study Population

This study is a retrospective cross-sectional analysis of a cohort of patients from the Functional Unit of Cerebrovascular Diseases at the Hospital Clinic of Barcelona, from June 2022 to November 2024, who experienced a lacunar ischemic stroke within the last month and underwent an investigational MRI protocol using a 3-Tesla MR scanner [13].

For this specific project, we excluded individuals with incomplete clinical data or insufficient imaging sequences, which were necessary for evaluating the primary research variables.

### 2.2. Clinical Information Collection and Definitions

Clinical reports from the ischemic events were the source of all clinical data. The data collected for each patient included sex, age, body mass index (BMI), blood pressure, biochemical parameters (total cholesterol, HDLc, LDLc, glycemia, and glycated hemoglobin [HbA1c]), and smoking.

The VRFs were defined as follows. Hypertension was defined as the presence of a diagnosis of hypertension at admission according to clinical records. Diabetes mellitus was defined as an HbA1c level ≥ 6.5% or a known diagnosis of diabetes according to clinical records. Hyperlipidemia was defined as a prior diagnosis of hyperlipidemia in clinical records. Finally, the smoking status was defined as currently smoking.

### 2.3. MRI Analysis

MRI data processing and lesion analysis were conducted at the MRI analysis facility of the IDIBAPS Biomedical Research Institute (Barcelona, Spain). The specific MRI sequences of each patient were thoroughly examined for the characterization of each of the four cSVD lesions of interest according to the STRIVE criteria [3]. Evaluation and manual segmentation of lacunes and CMBs were performed using ITK-snap software (www.itk.snap.org, accessed on 1 November 2024) [14]. Lacunes were identified at visual inspection, evaluating both FLAIR and T1-weighted sequences, and then segmented on the FLAIR sequences. CMBs were identified and segmented on susceptibility weighted image (SWI) sequences. WMH was segmented using an automated pipeline [15]. The subacute stroke lesion volume was removed after manual segmentation. PVSs were segmented using a technique based on using the 3D Frangi filter to enhance vessel-like structures [16]. We selected two main regions for PVS quantification: white matter (WM) and the basal ganglia (BG). All WMH and PVS segmentations were revised and edited for artifacts and inaccuracies if needed. Full information about the imaging protocol and MRI acquisition details is available elsewhere [17].

To enable visual assessment, after performing all individual lesion segmentations (WMH, PVSs, lacunes and CMBs), the lesion masks were spatially normalized to a standard brain template space (MNI). Subsequently, the normalized maps were averaged for each kind of lesion to obtain heat maps that represented the distribution of each cSVD segmentation in relation to the presence of each of the four pre-established VRFs. FreeSurfer (https://surfer.nmr.mgh.harvard.edu/, accessed on 1 December 2024) [18] was used to obtain brain subcortical parcellation, and the load of each of the imaging markers was assessed in specific brain regions (white matter, thalamus, brainstem, hippocampus, and basal ganglia).

### 2.4. Statistical Analysis

Means or medians were used for the central tendency, and standard deviation measures and interquartile ranges were used as dispersion measures according to each variable’s parametric or non-parametric features. Comparisons were analyzed using ANOVA and the Kruskal–Wallis test accordingly.

The main dependent variables were the WMH (fractional volume obtained from WMH volume/intracranial volume, expressed as a percentage), PVSs (fractional volume in the BG, WM, and all of the brain except the cortical structures, expressed as a percentage), and the number of CMBs and lacunes.

The main independent variables were a history of hypertension, hyperlipidemia, diabetes, and current smoking.

The relationships between each VRF and lacunes, as well as CMBs, were evaluated using logistic regression. To create comparable groups, these dependent variables were dichotomized according to their median values. Associations with continuous variables, such as PVSs and WMH, were assessed using linear regression models. Both the logistic and linear regression models were examined in multivariable analyses, including the 4 VRFs, and after adjusting for age and sex.

Afterward, we assessed each specific cSVD lesion spatial pattern through a visual qualitative examination of the heat maps and a volumetric comparison in specific brain areas. For this, we compared the regional volumes of each cSVD marker across various brain region defined by FreeSurfer’s anatomical territories, including the white matter, thalamus, basal ganglia, hippocampus, and brainstem. We calculated Pearson correlation coefficients to identify the regions with the highest involvement of cSVD markers for each VRF. Additionally, we adjusted the significance values obtained from the regional analysis for multiple comparisons using the Sidak method.

Statistical significance was set at *p* < 0.05 for all analyses, and all hypotheses were 2-sided. Analyses were performed in STATA v.15.

## 3. Results

All 93 patients screened from the cohort had complete data and usable MRIs for the analysis. The mean age was 70 years, and 25 (26.7%) subjects were female. A history of hypertension at admission, hyperlipidemia, diabetes, or smoking was common (61%, 46%, 24%, and 24%, respectively). Table 1 shows the complete patient demographic, clinical, and imaging features.

### 3.1. Hypertension

In the cohort, 61 (65.6%) patients had hypertension at admission, and 51 (54.8%) were on antihypertensive therapy. Patients with hypertension at admission were older, had lower cholesterol levels, and had higher glycemia and HbA1c levels (Table 2). In the univariable analysis, patients with hypertension had more lacunes (*p* < 0.001) but did not differ in their CMB amounts. Patients with hypertension revealed a greater volume of WMH (*p* = 0.018) and PVSs in the basal ganglia (*p* = 0.002) and white matter (*p* < 0.001) compared with the patients without hypertension.

**Figure 2:**
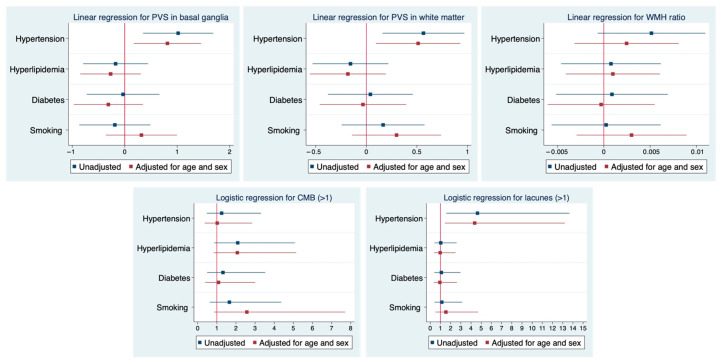
Associations between VRF and MRI markers of cSVD. Results from multivariable regression models. Forest plots illustrate the associations between cardiovascular risk factors (hypertension, hyperlipidemia, diabetes, and smoking) and neuroimaging markers of cSVD. Linear regression models were applied to assess the relationship with PVSs in the basal ganglia and white matter, as well as the WMH volume ratio. Logistic regression models were used for the presence of >1 CMB and >1 lacune. Blue markers indicate unadjusted estimates, and red markers indicate estimates adjusted for age and sex along with all the explored VRFs. Horizontal lines represent 95% confidence intervals. Abbreviations: WMH = white matter hyperintensity; PVS = perivascular space; CMB = cerebral microbleed.

**Figure 3:**
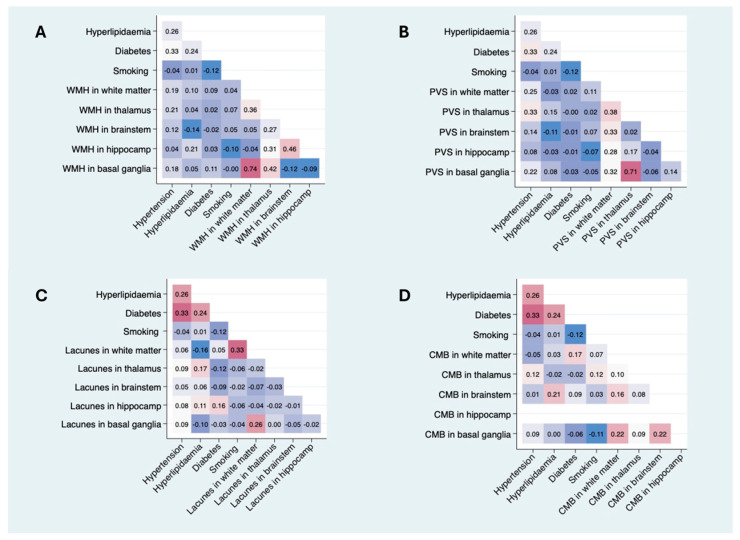
Pearson correlation matrices between VRFs and regional cSVD imaging markers. Each matrix displays the Pearson correlation coefficients between the presence of VRFs (hypertension, dyslipidemia, diabetes, and smoking) and the burden of imaging markers—WMH, PVSs, lacunes, and CMBs—across specific brain regions (white matter, thalamus, brainstem, hippocampus, and basal ganglia). (**A**) Pearson correlation matrix between VRFs and regional WMH volume. (**B**) Pearson correlation matrix between VRFs and regional PVS volumes. (**C**) Pearson correlation matrix between VRFs and regional lacune volumes. (**D**) Pearson correlation matrix between VRFs and regional CMB volumes. Color intensity represents the strength of correlation (blue = negative; red = positive). Only unadjusted bivariate correlations are shown. Abbreviations: WMH = white matter hyperintensity; PVS = perivascular space; CMB = cerebral microbleed; VRF = vascular risk factor. Comprehensive information on correlations is included in the Supplementary Materials.

**Figure 4:**
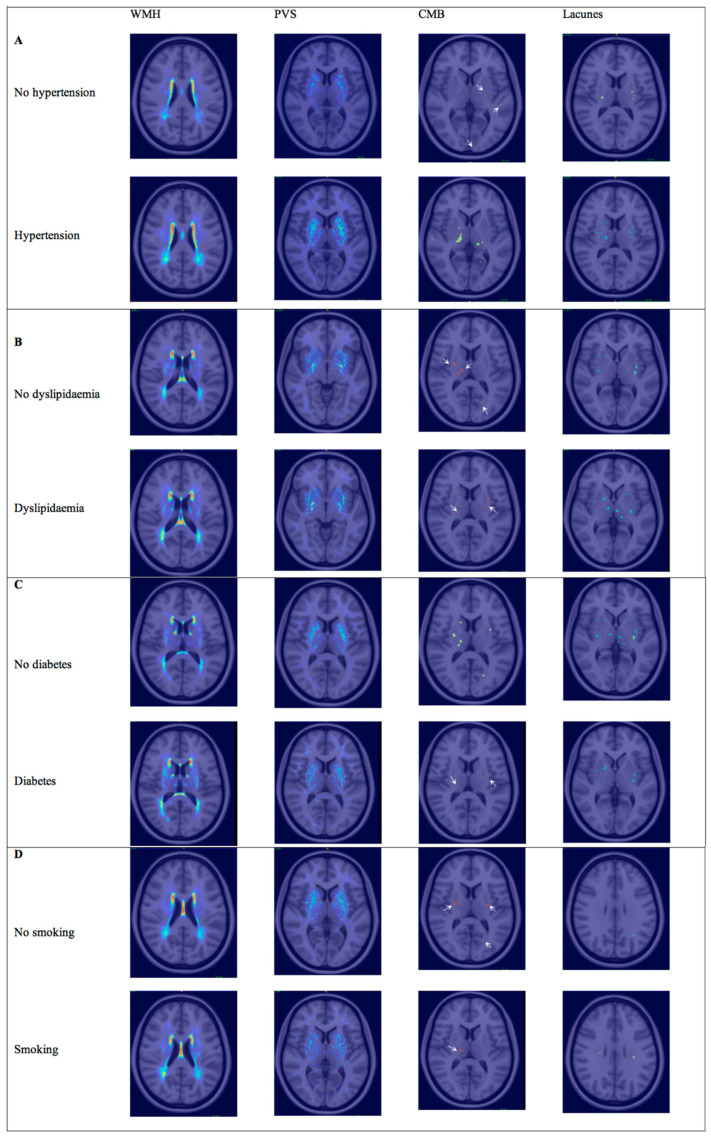
Lesion heat maps of cSVD marker distribution according to VRF. Spatial distribution of WMH, PVSs, lacunes, and CMBs in patients grouped by the presence or absence of each VRF. (**A**) Lesion heat maps for hypertension. (**B**) Lesion heat maps for hyperlipidemia. (**C**) Lesion heat maps for diabetes. (**D**) Lesion heat maps for smoking. Each heat map represents the cumulative burden and anatomical localization of lesions. Only representative brain slices are shown for each marker, selected to best illustrate the most affected regions. Warmer colors indicate higher lesion density. White arrows point out CMBs. Abbreviations: WMH = white matter hyperintensity; PVS = perivascular space; CMB = cerebral microbleed.

In the multivariable analysis, hypertension was still related to the PVS volume for both regions (basal ganglia: b = 0.8, *p* = 0.014; white matter: b = 0.5, *p* = 0.016) and lacunes (OR for lacunes > 1, (95% CI), 4.4 (1.4–13.2), *p* = 0.009), while the association with WMH was no longer significant (b = 0.002, *p* = 0.388) despite maintaining a positive trend (Figure 2). In the regional analysis (Figure 3), the correlations between hypertension and the WMH volumes were higher in the thalamus, white matter, and basal ganglia (Sidak *p* = 0.044, 0.070, and 0.089, respectively). The correlations with PVSs were higher in the thalamus, white matter, and basal ganglia (Sidak *p* = 0.001, 0.016, and 0.033, respectively). The correlations with lacunes and the CMB volume were low overall (r < 0.013). Comprehensive information on the correlations is included in the Supplementary Materials. Visual analysis of lesion heat maps (Figure 4A) revealed a predominance of posterior periventricular WMH patterns in the hypertensive group, along with an increased PVS volume in the anterior regions of the white matter and the brainstem. Additionally, both CMBs and lacunes appeared to be more frequent in deep brain regions among the hypertensive individuals, whereas in the non-hypertensive subjects, the lesions were more cortically distributed.

### 3.2. Hyperlipidemia

Forty-six patients had hyperlipidemia at admission in the cohort (49.5%), and only 33.3% were under hypolipidemic treatment. Patients with hyperlipidemia at admission had lower LDLc levels than those without hyperlipidemia, whereas HbA1c levels were higher in the hyperlipidemia group (*p* = 0.017). The patients with hyperlipidemia had more CMBs compared with those without (*p* = 0.020). In the multivariable analysis, hyperlipidemia did not show any significant associations with SVD markers under MRI, although a nearly significant positive trend was observed for a higher number of CMBs. In the regional analysis (Figure 3), the correlation between hyperlipidemia and WMH volumes was significant only in the hippocampus (Sidak *p* = 0.045).

The correlations with PVSs and lacunes were mild or low overall (r < 0.17). The correlation with the CMB volume was significant in the brainstem (Sidak *p* = 0.045). Although visual analysis of the lesion heat maps did not show evident differences in PVS and CMB distribution between groups, the patients with dyslipidemia appeared to show more lacunes in the thalamus and WMH in the hippocampus (Figure 4B).

### 3.3. Diabetes

Twenty-eight (30.1%) patients had diabetes, most of whom were treated with oral antidiabetic drugs (87.5%), and only 33.3% were treated with insulin. Twenty (21.5%) patients had no prior history of diabetes at the time of admission but did have HbA1c levels above 6.5%. The diabetic patients were older than the non-diabetic patients. Blood glucose levels and HbA1c levels were higher in the diabetic patients. The total cholesterol and LDLc levels were higher in the non-diabetic group. In the univariable analysis, the diabetic patients had more lacunes (*p* = 0.049) but did not show any significant associations with the SVD markers in the multivariable models. In the regional analysis (Figure 3), the correlations between diabetes and cSVD markers were mild or low overall (r < 0.17). In the visual analysis, the patients with diabetes exhibited a posterior periventricular WMH pattern and increased WMH in the basal ganglia, particularly the putamen. The PVSs seemed to follow an anterior distribution in the centrum semiovale. In contrast, both lacunes and CMBs appeared to be more visually prominent in the non-diabetic patients (Figure 4C).

### 3.4. Smoking

Twenty-four (25.8%) patients were current smokers. The total cholesterol and alcohol consumption were higher in the smoker group (*p* = 0.023 and *p* = 0.002, respectively). Smokers presented a nearly significant trend for more CMBs (*p* = 0.07) in the univariable analysis. No associations were found in the multivariable models, although smoking showed a positive trend for all SVD markers after adjusting for sex and age (OR, 95% CI: 2.6, 0.9–7.7; *p* = 0.092). In the regional analysis (Figure 3), there was a significant correlation between smoking and lacunes in the WM (Sidak *p* = 0.001), while the remaining associations were mild or low (r < 0.13). Visually, the lesion heat maps of the non-smokers appeared to present a higher overall PVS burden and more CMBs. While a greater amount of lacunes were found in the non-smokers, those located in white matter were more prominent in the smokers (Figure 4D).

## 4. Discussion

VRFs were greatly represented in our cohort, ranging from 65% for hypertension to 25% for smoking. Univariable associations with the global load of some of the cSVD markers were evident for each VRF, except for smoking, although they were mostly driven by concurrent hypertension in the multivariable analysis. The spatial analysis revealed that each risk factor was associated with a distinct topographical pattern of lesion distribution, suggesting different pathophysiological mechanisms affecting specific brain regions. Collectively, our results suggest that the impact of VRFs on cSVD is heterogeneous, both in terms of marker specificity and regional brain involvement.

As expected, the patients with hypertension were older, which represents a key confounder when assessing the relationships between VRFs and cSVD markers. In the global cSVD load analysis, only the PVS volume and number of lacunes remained significantly associated with hypertension after adjusting for age and sex. A non-significant trend was observed for the WMH volume, which was likely constrained by sample size and collinearity issues. Moreover, the regional correlation analysis showed significant associations between hypertension and PVS volumes in the thalamus, white matter, and basal ganglia, suggesting that hypertension may contribute to impaired perivascular dynamics, particularly in regions strategically vulnerable to hypertensive injury [19]. Additionally, upon visual heat map inspection, the PVS volumes appeared to be higher in anterior regions and the brainstem, which are both regions potentially vulnerable to high blood pressure gradients in hypertension [20].

Despite the non-significance in the multivariable analysis, the regional correlation analysis revealed that WMH volumes were more strongly associated with hypertension in the thalamus, white matter, and basal ganglia. This supports the strong pathogenic link between chronic blood pressure elevation and arteriosclerotic damage to deep perforating vessels. This process affects subcortical circuits and leads to lacunar infarcts and demyelination, which are visible as WMH [1,21]. These findings are consistent with large prospective studies, including the LADIS study and the Rotterdam Scan Study, which identified hypertension as the strongest modifiable determinant of both WMH and lacunes in the elderly population [22,23]. The visual analysis of the WMH distribution revealed a predominance of WMH in the posterior periventricular region, in contrast to the classic frontal periventricular pattern associated with long-standing blood pressure elevation [23]. One possible explanation for this finding is that previous studies have primarily compared the WMH distribution between different etiologies of cSVD, such as cerebral amyloid angiopathy and arteriosclerotic cSVD, rather than examining variability in lesion patterns within a single etiological group like hypertensive cSVD (type 1). Alternatively, the baseline differences in key cohort features between hypertensive and non-hypertensive patients, such as age, might have significantly impacted the WMH distribution. Finally, lacunes and CMBs were more frequent in deep locations among patients with hypertension, while the non-hypertensive individuals showed a more cortical distribution, again supporting a distinct hypertensive microvascular profile.

Patients with hyperlipidemia exhibited some unexpected characteristics, including lower LDL cholesterol levels compared with those without hyperlipidemia. This finding may reflect treatment effects or underdiagnoses. The observed correlation between hyperlipidemia and higher HbA1c levels suggests insulin resistance or metabolic syndrome, both of which increase the risk of microvascular damage.

Neuroimaging analysis revealed that the patients with hyperlipidemia had a higher number of CMBs in the univariable analysis. This finding was nearly statistically significant in the multivariable analysis, including all VRFs and after adjusting for sex and age, likely due to the small sample size and the influence of age-related amyloid deposition. Regional correlation analysis further supported this association, identifying a significant link between hyperlipidemia and CMB volume, specifically in the brainstem. This finding is consistent with previous studies suggesting that altered lipid metabolism may increase vascular fragility [24], as low cholesterol levels may paradoxically increase the risk of CMBs due to compromising the integrity of the blood–brain barrier [25].

Interestingly, the WMH volume was significantly associated with hyperlipidemia in the hippocampus, suggesting a possible role of lipid-related perfusion or neuroinflammatory mechanisms in this region. Recent evidence from the UK Biobank cohort supports this interpretation. Jiang et al. identified a blood-based lipid profile associated with reduced hippocampal volume and altered resting-state brain activation in obese adults, highlighting the hippocampus as a region vulnerable to metabolic and vascular disturbances [26].

The heat map examination partially aligned with these results; no clear differences were observed in the spatial distribution of PVSs and CMBs, but lacunes were notably more frequent in the thalamus among patients with hyperlipidemia. This may indicate increased vulnerability of deep gray matter structures to lipid-related endothelial dysfunction. Moreover, WMH volumes in the hippocampus were mildly higher, resonating with the findings from the UK Biobank.

In the univariable analysis, diabetic patients exhibited more lacunes and a greater burden of PVSs in the basal ganglia, along with a trend toward increased WMH volumes. However, these associations disappeared after multivariable adjustment, which contrasts with the known microangiopathic nature of diabetes, implying that oxidative stress triggered by advanced glycation end products (AGEs) leads to endothelial dysfunction and impaired cerebral autoregulation [11]. This result could be explained by the coexistence of other VRFs and the overall good glycemic control in our cohort.

In the regional analysis, correlations between diabetes and cSVD markers were also mild overall and did not reach statistical significance. However, the visual heat map analysis provided some interesting findings. Patients with diabetes had a posterior periventricular WMH pattern, similar to hypertensive individuals, and showed a higher WMH volume in the basal ganglia, particularly the putamen. Moreover, the PVSs in the diabetic group appeared to follow a more anterior distribution in the centrum semiovale, similar to the results reported by other authors [27], who suggested a potential intermediary role of perivascular dysfunction in diabetes-related cSVD. Interestingly, both lacunes and CMBs were visually more abundant in the non-diabetic group, potentially due to the overlapping effects of other VRFs, sampling variability, or treatment and lifestyle changes addressing diabetic patients.

As expected, the smokers were younger, consumed more alcohol, and had higher cholesterol levels. Although most associations between smoking and cSVD markers did not reach statistical significance, the smokers presented a trend for all SVD markers, which was nearly significant for CMBs, and the regional analysis identified a significant correlation between smoking and the number of lacunes in the white matter. This finding supports the hypothesis that tobacco toxins, like nicotine, carbon monoxide, polycyclic aromatic hydrocarbons, and heavy metals, cause endothelial damage via oxidative stress and inflammation. This damage can lead to thrombotic events or serious impairment of the blood–brain barrier, resulting in the leaking of blood components [28]. Other correlations, including those with WMH and PVSs, were generally weak, suggesting that the impact of smoking may be more selective for single vessel events rather than diffuse myelin involvement in the white matter. However, large prospective studies, such as the Framingham Study or that of Hara M. et al., showed that smoking, among other VRFs, was independently associated with increased WMH volumes, suggesting the cumulative effect of smoking on chronic small vessel injury [29,30].

The lesion heat maps provided a complementary understanding; non-smokers displayed a greater overall PVS and CMB burden, likely reflecting subgroup sampling imbalance (only 25% were smokers), due to their older ages and associated comorbidities. However, white matter lacunes were more prevalent in the smokers, reinforcing the idea that tobacco exposure may selectively affect the small penetrating arteries that supply the deep white matter.

This study has notable strengths. First, the imaging protocol utilized a 3T MRI scanner, adhering to current recommendations for cSVD research. Second, we employed objective measures of cSVD burden through validated segmentation methods, which were further refined by manual correction. Furthermore, the visual analysis complemented the quantitative findings by highlighting region-specific patterns of lesion distribution, offering an additional spatial perspective beyond volumetric differences alone.

However, this study presents limitations. Due to the retrospective design of the study, we were unable to assess causality or the progression of SVD markers. Additionally, data on the duration of vascular risk factors and treatment adherence were not systematically collected, rendering the information unreliable. Patients with unknown hypertension or diabetes may have been incorrectly classified; however, this percentage was likely negligible, as the overall prevalence of these risk factors in this study aligns with the expected prevalence for this population. Furthermore, blood pressure tends to be elevated during the acute phase of stroke, and therefore, this data was not used to define a new diagnosis of hypertension. Likewise, due to statin treatment, cholesterol levels were lower in the patients with a known diagnosis of hyperlipidemia compared with those without it. Therefore, cholesterol levels were not used to classify patients as having or not having hyperlipidemia. The relatively small sample size may have limited the statistical power for detecting subtle associations, particularly in the multivariate analyses. Nevertheless, this is partially mitigated by the cohort’s homogeneity in clinical characteristics and the consistency of the imaging protocol. Additionally, we analyzed the influence of each VRF individually, although a clinically significant cumulative effect of VRFs may occur, which is a common phenomenon. Despite providing valuable insights into the spatial distribution of lesions, visual analysis has limitations in accuracy and reproducibility, as it relies on subjective interpretation and may be less sensitive to subtle differences. Unfortunately, the limited sample size did not allow for a more precise analysis using quantitative regional assessment based on voxel-wise comparisons.

Future studies, including larger cohorts that allow for more robust analysis of confounding and interaction effects between variables and more precise spatial analyses, would be helpful in better understanding how each VRF interacts with and collectively contributes to the overall burden of cSVD.

## 5. Conclusions

This study provides further evidence that common modifiable VRFs such as hypertension, hyperlipidemia, diabetes, and smoking contribute differently to the burden of cSVD on brain MRI. Among all of these, hypertension showed the strongest and most consistent association, especially with lacunes and PVSs. The observed patterns suggest that each VRF may exert a distinct pathological effect on small cerebral vessels, potentially through different mechanisms, such as arteriosclerosis, impaired clearance, or endothelial dysfunction.

The overall findings support the hypothesis that cSVD is a heterogeneous condition, with imaging phenotypes shaped by underlying risk factors. The study results reinforce the need for personalized approaches to prevention and risk factor management in cSVD, as well as the importance of further research into the cumulative and interactive effects of multiple VRFs on cSVD progression. Larger studies combining longitudinal follow-up and integrative analysis models are needed to better understand and clarify the mechanisms underlying regional susceptibility to vascular damage and lesion heterogeneity in cSVD.

## Figures and Tables

**Table 1:** Clinical, demographic, and radiological characteristics of the study population in VRF subgroups.

Clinical Characteristics	Patients (n = 93)
Age (years), mean (SD)	70.2 (10.8)
Female sex, n (%)	25 (26.7)
BMI (kg/m^2^), median (SD)	27.2 (4.0)
History of hypertension, n (%)	61 (65.6)
ACEI, n (%)	48 (51.2)
ARBs, n (%)	19 (20.4)
Calcium channel blockers, n (%)	17 (18.3)
Beta blockers, n (%)	10 (10.8)
Diuretics, n (%)	17 (18.3)
History of hyperlipidemia, n (%)	46 (49.5)
Total cholesterol (mg/dL), mean (SD)	186 (47.0)
LDLc (mg/dL), mean (SD)	117.9 (40.3)
HDLc (mg/dL), mean (SD)	43.2 (13.2)
History of diabetes mellitus at admission, n (%)	28 (30.1)
Glycemia (mg/dL), mean (SD)	120.1 (35)
HbA1c (%), mean (SD)	6 (1.1)
HbA1c ≥ 6.5%, n (%)	20 (21.5)
Current smoker, n (%)	24 (25.8)
Any alcohol intake, n (%)	29 (31.2)
Lacunes, presence of, n (%)	1.9 (2.5)
Lacunes, number of, median (IQR)	1 (0–3)
Patients with >1 lacune, n (%)	38 (48.9)
CMB, presence of, n (%)	18 (25.4)
CMB, number, median (IQR)	1 (0–4)
Patients with >1 CMB, n (%)	39 (41.9)
BG-PVS (%), mean (SD)	3.8 (1.5)
WM-PVS (%), mean (SD)	1.1 (1.1)
WM-ratio (WMH/ICV), median (IQR)	0.7 (0.4–1.7)

Abbreviations: SD = standard deviation; IQR = interquartile rank; BMI = body mass index; ACEI = angiotensin-converting enzyme inhibitors; ARBs = angiotensin receptor blockers; LDLc = low-density lipoprotein cholesterol; HDLc = high-density lipoprotein cholesterol; HbA1c = glycated hemoglobin; CMB = cerebral microbleed; PVS = perivascular space; BG = basal ganglia; WM = white matter; WMH = white matter hyperintensity; ICV = intracranial volume.

**Table 2:** Comparison of clinical variables by vascular risk factor.

Variable	Hypertension			Hyperlipidemia			Diabetes Mellitus			Smoking		
	No	Yes	***p***	No	Yes	***p***	No	Yes	***p***	No	Yes	***p***
**Clinical Characteristics**												
Age, mean (SD)	66.0 (11.9)	72.3 (9.5)	**0.011**	68.6 (12.2)	71.8 (9.0)	0.150	68.0 (11.2)	75.2 (8.0)	**<0.001**	72.5 (10.2)	63.3 (9.5)	**<0.001**
BMI (kg/m^2^), mean (SD)	26.5 (4.3)	27.6 (3.9)	0.120	27.8 (4.7)	26.7 (3.2)	0.280	27.2 (4.3)	27.4 (3.3)	0.740	27.5 (4.0)	26.6 (4.0)	0.290
Total cholesterol (mg/dL), mean (SD)	195.5 (33.3)	180.9 (52.6)	**0.030**	191.0 (32.9)	180.8 (58.1)	0.080	195.0 (48.6)	164.1 (35.2)	**0.003**	179.5 (38.6)	208.4 (65.2)	**0.023**
LDLc (mg/dL), mean (SD)	125.9 (27.51)	113.6 (45.37)	**0.020**	123.8 (28.4)	111.8 (49.3)	**0.030**	125.8 (41.0)	98.7 (31.9)	**<0.001**	113.1 (35.8)	134.4 (50.8)	0.052
HDLc (mg/dL), mean (SD)	46.0 (14.5)	41.8 (12.3)	0.120	45.0 (15.4)	41.5 (10.4)	0.440	44.7 (14.2)	39.9 (9.6)	0.160	44.3 (12.8)	39.7 (14.1)	0.054
Glycemia (mg/dL), mean (SD)	109.5 (24.6)	125.7 (38.4)	**0.030**	114.7 (30.0)	125.6 (39.0)	0.180	106.8 (18.3)	151.1 (44.3)	**<0.001**	120.5 (36.1)	119.1 (32.3)	0.980
HbA1c (%), mean (SD)	5.6 (0.6)	6.2 (1.2)	**0.005**	5.8 (0.7)	6.3 (1.3)	**0.017**	5.6 (0.5)	7.1 (1.3)	**<0.001**	6.1 (1.2)	6.0 (0.9)	0.870
Any alcohol intake, n (%)	9 (28)	20 (32.8)	0.820	16 (34.0)	13 (28.3)	0.710	24 (36.9)	5 (17.9)	0.110	15 (21.7)	14 (58.3)	**0.002**
**Neuroimaging Markers**												
Lacunes, presence of, n (%)	14.0 (43.8)	47.0 (77.0)	**0.003**	27.0 (57.4)	34.0 (73.9)	0.150	38.0 (58.5)	23.0 (82.1)	**0.049**	43.0 (62.3)	18.0 (75.0)	0.380
Lacunes, number of, median (IQR)	0.0 (0–1)	2.0 (1–4)	**<0.001**	1.0 (0–3)	1.0 (0.3–4)	0.110	1.0 (0–3)	1.5 (1–4.3)	0.061	1.0 (0–3)	1.0 (0.8–3)	0.350
CMBs, presence of, n (%)	18.0 (56.2)	41.0 (67.2)	0.410	27.0 (56.2)	32.0 (71.7)	**0.020**	39.0 (59.1)	20.0 (74.1)	0.260	41.0 (58.6)	18.0 (78.3)	0.150
CMBs, number, median (IQR)	1.0 (0–3)	1.0 (0–4)	0.660	1.0 (0–2.5)	2.0 (0–4.8)	**0.020**	1.0 (0–3)	1.5 (0.8–4.3)	0.190	1.0 (0–3)	1.5 (0–5)	0.070
BG-PVS (%), mean (SD)	3.1 (1.4)	4.1 (1.4)	**0.002**	3.7 (1.6)	3.8 (1.3)	0.810	3.7 (1.6)	4.0 (1.3)	0.390	3.8 (1.3)	3.6 (1.9)	0.380
WM-PVS (%), mean (SD)	0.8 (0.8)	1.3 (0.9)	**<0.001**	1.1 (1.0)	1.1 (0.8)	0.440	1.1 (0.9)	1.2 (0.9)	0.190	1.1 (0.8)	1.2 (1.1)	0.950
WMH ratio, mean (SD)	0.011 (0.0093)	0.017 (0.013)	**0.018**	0.014 (0.01)	0.016 (0.014)	0.530	0.014 (0.011)	0.017 (0.015)	0.390	0.015 (0.013)	0.015 (0.012)	0.900

Abbreviations: SD = standard deviation; IQR = interquartile rank; *p* = statistical significance; BMI = body mass index; LDLc = low-density lipoprotein cholesterol; HDLc = high-density lipoprotein cholesterol; HbA1c = glycated hemoglobin; CMB = cerebral microbleed; PVS = perivascular space; BG = basal ganglia; WM = white matter; WMH = white matter hyperintensity.

## Data Availability

The original contributions presented in this study are included in the article and Supplementary Materials. Further inquiries can be directed toward the corresponding author.

## Supplementary Materials

The following supporting information can be downloaded at: https://www.mdpi.com/article/10.3390/brainsci15080804/s1, Tables S1–S4: Pearson Correlations Between Cardiovascular Risk Factors and MRI Markers of Cerebral Small Vessel Disease by Brain Region.
