# Supplementary material for: Differences in Cerebral Small Vessel Disease Magnetic Resonance Imaging Depending on Cardiovascular Risk Factors: A Retrospective Cross-Sectional Study
Source: Brain Sci. 2025 Jul 28;15(8):804. doi: 10.3390/brainsci15080804 (PMC12384560; doi:10.3390/brainsci15080804)
Supplement: Supplementary file 1 [file brainsci-15-00804-s001.zip › brainsci-3748284-supplementary.pdf]

Supplementary Material

Supplementary Table S1-S4: Pearson Correlations Between Cardiovascular Risk Factors and MRI Markers of Cerebral Small Vessel Disease by Brain Region.

Table S1: Small Vessel Disease Marker: Perivascular Spaces by Brain Region.

	Hypertension<br>Pearson's coefficient<br>(Sidak *p*)	Hyperlipidemia<br>Pearson's coefficient<br>(Sidak *p*)	Diabetes<br>Pearson's coefficient<br>(Sidak *p*)	Smoking<br>Pearson's coefficient<br>(Sidak *p*)
**PVS in white matter**	0.249<br>(0.439)	-0.033<br>(1.000)	0.017<br>(1.000)	0.109<br>(1.000)
**PVS in thalamus**	**0.320**<br>**(0.045)**	0.151<br>(0.997)	-0.003<br>(1.000)	0.025<br>(1.000)
**PVS in brainstem**	0.141<br>(0.999)	-0.112<br>(1.000)	0.012<br>(1.000)	0.074<br>(1.000)
**PVS in hippocamp**	0.076<br>(1.000)	-0.027<br>(1.000)	-0.013<br>(1.000)	-0.072<br>(1.000)
**PVS in basal ganglia**	0.221<br>(0.703)	0.080<br>(1.000)	-0.032<br>(1.000)	-0.052<br>(1.000)

PVS: Perivascular Spaces

Table S2: Small Vessel Disease Marker: White Matter Hyperintensity by Brain Region.

	Hypertension<br>Pearson's coefficient<br>(Sidak *p*)	Hyperlipidemia<br>Pearson's coefficient<br>(Sidak *p*)	Diabetes<br>Pearson's coefficient<br>(Sidak *p*)	Smoking<br>Pearson's coefficient<br>(Sidak *p*)
**WMH in white matter**	0.189<br>(0.926)	0.096<br>(1.000)	0.093<br>(1.000)	0.038<br>(1.000)
**WMH in thalamus**	0.209<br>(0.803)	0.040<br>(1.000)	0.022<br>(1.000)	0.070<br>(1.000)
**WMH in brainstem**	0.116<br>(1.000)	-0.139<br>(1.000)	-0.015<br>(1.000)	0.055<br>(1.000)
**WMH in hippocamp**	0.041<br>(1.000)	0.209<br>(0.808)	0.025<br>(1.000)	-0.104<br>(1.000)
**WMH in basal ganglia**	0.177<br>(0.965)	0.46<br>(1.000)	0.110<br>(1.000)	-0.003<br>(1.000)

WMH: White Matter Hyperintensity

Table S3: Small Vessel Disease Marker: Lacunes by Brain Region.

	Hypertension<br>Pearson's coefficient<br>(Sidak *p*)	Hyperlipidemia<br>Pearson's coefficient<br>(Sidak *p*)	Diabetes<br>Pearson's coefficient<br>(Sidak *p*)	Smoking<br>Pearson's coefficient<br>(Sidak *p*)
**Lacunes in white matter**	0.057<br>(1.000)	-0.162<br>(0.991)	0.047<br>(1.000)	**0.330**<br>**(0.044)**
**Lacunes in thalamus**	0.095<br>(1.000)	0.166<br>(0.986)	-0.116<br>(1.000)	-0.060<br>(1.000)

**Lacunes in brainstem**	0.046<br>(1.000)	0.058<br>(1.000)	-0.094<br>(1.000)	-0.022<br>(1.000)
**Lacunes in hippocamp**	0.041<br>(1.000)	0.105<br>(1.000)	0.159<br>(0.993)	-0.062<br>(1.000)
**Lacunes in basal ganglia**	0.088<br>(1.000)	-0.101<br>(1.000)	-0.028<br>(1.000)	-0.037<br>(1.000)

Table S4: Small Vessel Disease Marker: Cerebral Microbleeds by Brain Region.

	**Hypertension**<br>Pearson's coefficient<br>(Sidak *p*)	**Hyperlipidemia**<br>Pearson's coefficient<br>(Sidak *p*)	**Diabetes**<br>Pearson's coefficient<br>(Sidak *p*)	**Smoking**<br>Pearson's coefficient<br>(Sidak *p*)
**CMB in white matter**	0.050<br>(1.000)	0.033<br>(1.000)	0.167<br>(0.985)	0.071<br>(1.000)
**CMB in thalamus**	0.120<br>(1.000)	-0.021<br>(1.000)	-0.021<br>(1.000)	0.120<br>(1.000)
**CMB in brainstem**	0.012<br>(1.000)	0.208<br>(0.811)	0.088<br>(1.000)	0.028<br>(1.000)
**CMB in basal ganglia**	0.088<br>(1.000)	0.001<br>(1.000)	-0.062<br>(1.000)	-0.108<br>(1.000)
**CMB in white matter**	0.050<br>(1.000)	0.033<br>(1.000)	0.167<br>(0.985)	0.071<br>(1.000)

CMB: Cerebral Microbleeds
